# Personality Type D, Level of Perceived Stress, Insomnia, and Depression Among High School Teachers in Poland

**DOI:** 10.3389/fpsyg.2021.626945

**Published:** 2021-09-21

**Authors:** Joanna Domagalska, Monika Rusin, Mehdi Razzaghi, Przemysław Nowak

**Affiliations:** ^1^Department of Environmental Health, Faculty of Health Sciences in Bytom, Medical University of Silesia, Katowice, Poland; ^2^Department of Mathematics, Bloomsburg University, Bloomsburg, PA, United States; ^3^Department of Pharmacology, Faculty of Medicine, University of Opole, Opole, Poland

**Keywords:** teachers, personality type D, stress, depression, insomnia

## Abstract

Teaching is inherently connected with specific burdens that may imply stressful situations. The goal of this study was to explore the prevalence of type D (distressed) personality in teachers. This is known to cause depressive episodes and sleep disorders, which not only have direct physical health effects, but can also impact the wellbeing of individuals and hence adversely affect their job performance. The participants consisted of 412 high school teachers from the Silesian Province, located in the south of Poland. Using the following research tools: Type D Scale (DS14), Perceived Stress Scale, Athens Insomnia Scale, and Beck Depression Inventory, it was found that type D personality was observed in a large percentage of teachers (30.1%). It was reported that teachers with distressed personality suffered from insomnia and depression significantly more often. Findings from the current study indicate the need to implement preventive activities focused on reducing psychosocial risk factors in the work environment in order to reduce the frequency of depressive disorders among teachers.

## Introduction

The concept of type D personality (distressed personality) was introduced in the scientific literature around 1996 by clinical psychologist Johan Denollet of the University of Tilburg as a result of identification of personality risk factors for cardiovascular diseases, in particular their progression and mortality (Denollet et al., 1996). This is a specific type of personality characterized by two elements (dimensions): negative affectivity and social inhibition. People with this type of personality tend to experience negative emotions – depression, anxiety, anger or hostility, negative self-perception, and usually report numerous complaints about psychosomatic disorders. They also present a tendency to social withdrawal, avoiding the potential threat resulting from social interactions, mainly because of fear of disapproval or rejection by other members of society. The type D personality construct is based on positive and negative emotionality, which are a manifestation of two main personality traits: neuroticism and introversion (De Fruyt and Denollet, 2002).

Scientific literature indicates that individuals with type D personality are four times more likely to develop coronary heart disease than those who do not. In addition, such people are characterized by a higher mortality rate due to cardiovascular diseases (Denollet and Pedersen, 2008). It is worth emphasizing that the relationship between distressed personality and coronary artery disease, multiple sclerosis, periodontal disease, hearing problems, diabetes, migraine, ulcerative colitis, chronic pain, cancer and gastric ulcer, and duodenum or skin diseases, including psoriasis, has frequently been confirmed in the past (Denollet et al., 1996; Mols and Denollet, 2010; Svansdottir et al., 2012; Condén et al., 2013b; Oginska-Bulik, 2014). Personality type D also predisposes to take risky health behaviors, including the use of destructive, unique stress coping strategies which indirectly determine reduced life satisfaction (Nefs et al., 2015; Conti et al., 2016; Mikula et al., 2018; Mizutani et al., 2018; Talaei-Khoei et al., 2018). For people with distressed personality, the course of the disease is a challenge because they experience anxiety and depression more often (De Fruyt and Denollet, 2002), subjectively assess their mental and physical condition as inferior, and the quality of life as lower than people with personality non-D (Sung et al., 2015; Zhang et al., 2016). Current scientific research also emphasizes changes in the regulation of the hypothalamic-pituitary-adrenal axis in people with type D personality (Habra et al., 2003). In these studies, negative emotionality and social inhibition are associated with excessive cortisol secretion in a stressful situation and during daily activities. High cortisol levels can be an important mediator between type D personality and an increased risk of coronary artery disease (Habra et al., 2003). Studies also confirm that type D is positively correlated with experiencing stress in the workplace (Mols and Denollet, 2010) and less frequent returns to work after a period of absence (De Fruyt and Denollet, 2002). The harmfulness of stress results from its permanent duration. Chronic exposure to stress leads to serious somatic diseases (arterial hypertension, myocardial infarction, neck muscle pain syndromes, peptic ulcer disease, decreased immunity, and cancer) or mental health (anxiety disorders and depression). It has been indicated that stress is a predictor of ischemic heart disease, some dermatological and musculoskeletal disorders, neurological and metabolic disorders, and sexual dysfunction (Schönfeld et al., 2016; Toussaint et al., 2016; Yaribeygi et al., 2017). Scientific research suggests that type D personality may be inherited (52%). The probability of inheriting negative affectivity is 46% and for social inhibition – 50% (Kupper et al., 2007).

Insomnia and depression are two major threats to current public health. The problem is even more apparent in the case of social professionals, including teachers. Sleep disturbances are common societal problems which unfortunately are largely overlooked. It is estimated that they occur in more than half of the adult population (Ribeiro Do Valle et al., 2013). Insomnia is one of the most common and most serious sleep disorders. It manifests as difficulty in falling asleep and maintaining sleep, waking up during the night and difficulty of re-entering to sleep or feeling excessive drowsiness after waking up (Glogowska-Gruszka and Josko-Ochojska, 2014; Pereira et al., 2014).

Insomnia is classified as a disruption in the amount, quality, and duration of sleep. Studies in Western Europe and the United States show that one-third of the adult population experiences insomnia symptoms. Studies from countries across the world have reported a prevalence of sleep problems ranging from 1.6 to 56.0%. However, the clinical diagnosis of insomnia is less frequent and ranges between 6 and 10% (Mayer et al., 2011; Koyanagi and Stickley, 2015). Sleep problems worsen with age, especially after the age of 40. They are more common (even twice) in women than in men. According to many epidemiological studies, risk factors include socioeconomic conditions, gender, age, lifestyle, occupation, level of burnout, and psychoactive substances (tobacco, drugs, and other substances used in the treatment of pain, anxiety, and depression; Taylor et al., 2005; Rosekind and Gregory, 2010).

The most common causes of insomnia are mental disorders including depressive episodes, psychoses, anxiety disorders, and personality disorders. Their occurrence is justified by emotional excitation, disorders of synaptic transmission, or biological rhythms. Chronic insomnia has a very adverse effect on the functioning of the whole body. Symptoms of long-term insomnia include fatigue, impaired mental and physical activity, impaired concentration, social functioning, and mood. Irritability, drowsiness, a lack of initiative skills, increased distraction, and a tendency to make mistakes are also common. The occurrence of insomnia may be manifested by tension headaches, a feeling of anxiety and excessive worry, and digestive tract malfunction (Krueger and Friedman, 2009; Hagg et al., 2015; Qaseem et al., 2016). In addition, untreated chronic insomnia can also contribute to the development of metabolic diseases, such as diabetes and obesity (Hagg et al., 2015; Bathgate et al., 2016; Qaseem et al., 2016).

The occurrence of insomnia is correlated with depression. Symptoms of depression may be psychological, physical, or social and may be manifested by sadness, changes in the structure of sleep, unexplained pain, lack of energy, reduced productivity, and efficiency at work or attempted suicide (World Health Organization, 2002; Sassarini, 2016). This is a very general classification of symptoms, and these are some of the many possible symptoms of the disease. Detailed diagnostic criteria are included in the current classification of ICD-10 and DSM V.

Depression is classified as a civilization disease. More than 264 million people affected worldwide suffer from depression [GBD (2017) Disease and Injury Incidence and Prevalence Collaborators, 2018]. It is anticipated that depression will be the second most-diagnosed disease on a global scale after circulatory failure (World Health Organization, 2017). It can affect anyone regardless of age. However, the largest number of cases concerns people between the ages of 55 and 74 (World Health Organization, 2017). Depression is twice as common in women than men (Sassarini, 2016). Depression also occurs among children and adolescents under 15years old, but this phenomenon is not as common as in older age groups. The total estimated number of people living with depression increased by 18.4% between 2005 and 2015 (GBD, 2015), reflecting the overall increase in the global population.

Researchers dealing with the problem of depressive disorders in an interdisciplinary aspect indicate stressogenicity of some professions as a predisposing factor to a higher frequency of this type of disease (e.g., health care workers, social workers, firefighters, policemen, accountants, cashiers, and teachers). It is indicated that over 40% of teachers resign from work during the first 5years of work because of experiencing depressive thoughts related to the sense of responsibility for the education of their students (Hussey et al., 2012).

Given the clinical relevance of findings on type D personality research in the context of adverse health effects, it is important to assess the potential relevance of that construct among apparently healthy people from the general population including teachers.

The aim of the study was to identify the relationship between type D personality, the level of perceived stress, and the occurrence of insomnia and depression symptoms among high school teachers in the Silesia Province in Poland.

## Materials and Methods

### Characteristics of the Participants

The survey (paper-based questionnaires) was conducted among 605 teachers of high schools from southern Poland, from the cities of Siemianowice Śląskie, Katowice, Chorzów, Bytom, Zabrze, and Ruda Śląska in the period from April to June 2016. After excluding incomplete and incorrect responses, 412 questionnaires were analyzed. The survey response rate was 68.09%.

### Characteristics of Research Tools

The presence of type D personality was determined using the Polish version of the 14-item type D scale (DS-14; Denollet, 2005; Oginska-Bulik et al., 2012). The scale consists of two 7-item subscales (negative affectivity and social inhibition). The DS-14 comprises two reliable subscales with seven items each for negative affectivity and social inhibition, rated on a 5-point Likert scale (from 0 false to 4 true). Type D personality is defined by a cutoff score of ≥10 on both subscales. The opposite of type D is non-type D (the results of both dimensions are below 10 points). Getting 10 points or more in one dimension qualify people as intermediate type. Due to the purpose of the work, the study took into account only the division into type D, intermediate, and non-type D personality. The internal consistency of the Polish version of the DS14 was good with Cronbach’s alpha of 0.86 for negative affectivity and 0.84 for social inhibition (Oginska-Bulik et al., 2012).

To assess the level of perceived stress, the Polish version (in adaption of Juczynski and Oginska-Bulik) of the perceived stress scale (PSS-10) questionnaire – Perceived Stress Scale by S. Cohen, T. Kamarck, and R. Marmelstein was used (Cohen et al., 1983; Oginska-Bulik et al., 2012). This tool is made up of 10 questions about thoughts and feelings related to experiences in the last month. Perceived stress scale is comprised of six negatively stated items (item 1, 2, 3, 6, 9, and 10) that measure individual perceived stress and four positively stated items (item 4, 5, 7, and 8) that measure individual coping ability. Respondents rated the frequency of occurrence for each item during the previous month based on a 5-point Likert scale that ranged from 0 (never) to 4 (very often). Perceived stress scale scores are obtained by summing across all items after reversing the scores of the four positively stated items. Total scores of PSS-10 ranged from 0 to 40 and higher scores indicate greater individual perceived stress. The general index (raw results; quotient scale) is transformed into standardized units (stens; interval scale) and is interpreted according to the properties characterizing the sten scale. In addition, sten results are further categorized. Sten scores within 1–4 are defined as low, scores of 5 and 6 are treated as average, while sten scores of 7–10 are considered as high (Oginska-Bulik et al., 2012). The Cronbach’s alpha for the Polish version of the scale is 0.86 (Oginska-Bulik et al., 2012). The third research tool was the Athens Insomnia Scale (AIS), which is one of the most commonly used scales, both for diagnostic purposes and in studies on the effectiveness of insomnia treatment. The questionnaire was created by a group of Greek scientists in 2000 (Soldatos et al., 2000). Its Polish version was translated and adopted by Warsaw researcher Fornal-Pawlowska. Athens insomnia scale is a short, eight-statement scale that allows quantitative measurement of insomnia symptoms based on the ICD-10 criteria. Respondents use Likert-type scales to show how severely certain sleep difficulties have affected them during the past month. The first five items pertain to sleep induction, awakenings during the night, final awakening, total sleep duration, and sleep quality. The last three refer to wellbeing, functional capacity, and sleepiness during the day. Each item is scored on a scale ranging from 0 (meaning that the item in question has not been a problem) to 3 (indicating more acute sleep difficulties). The total score is the sum of the scores and is between 0 and 24, where a higher score means worse sleep quality. Subjects with AIS scores of <6 can be reliably considered as not having insomnia (Soldatos et al., 2000; Fornal-Pawlowska et al., 2011). The Cronbach’s alpha for the Polish version of the scale is 0.90 (Fornal-Pawlowska et al., 2011).

To measure characteristic attitudes and symptoms of depression, a questionnaire developed by A. Beck and co-workers – the Beck Depression Inventory was used (Beck et al., 1961, 1988). Its Polish version was translated and adopted by Parnowski and Jernajczyk (1977). It consists of 21 items about symptoms of depression, cognitions, and physical symptoms. Each answer being scored on a scale ranging from 0 (symptom does not exist) to 3 (greatest symptom severity). It assesses depressive symptoms with high scores reflecting a greater severity of depressed mood (range = 0–63). Higher scores indicate greater symptom severity. In this study, a dichotomous division was used, with the distinction between depression (≥ 10 points) or no depressive symptoms (from 0 to 9 points) to separate teachers who were completely healthy from those who showed any symptoms of depression. The Cronbach’s alpha for this scale is 0.89.

### Statistical Analysis

Statistical analysis was performed by using the Statistica 12.0 (StatSoft Polska, Krakow, Poland) and SAS 9.2 (Institute Inc. Cary, United States). The Shapiro–Wilk test was used to assess the normality of continuous variables. The frequency (qualitative variables) is presented as percentage and total number of cases is shown by *N*. The Chi square test was used for testing independence of attributes in categorical variables and for detecting statistically significant differences between variables (in groups and subgroups). Insomnia and depression were defined as dichotomous variables based on absence or presence of the state. The main independent variables were the type of personality and level of perceived stress. Both were described as categorical variable with three levels. The influence of independent variables on the dependent variable was assessed using simple logistic regression analysis, the results of which are presented as raw odds ratio values. Statistical significance was set at a value of *p* less than 0.05.

## Results

The study participants consisted of 412 high school teachers from the Silesian Province in Poland, including 71.1% (*N*=293) women and 28.9% (*N*=119) men. The sample was representative of the population of Polish high school teachers. The most numerous group of teachers were respondents aged 36–45 (*N*=160; 38.8%). The surveyed population of teachers was dominated by people who had worked in the profession for over 20years (*N*=163; 39.5%). The smallest number of respondents had less than 5years of work experience (7.8% – *N*=32). The majority of teachers worked for more than one full-time employment (regular standard working hours – 18h per week; 1h – 45min), but less than 1.5 full-time jobs (*N*=152; 36.9%). A slightly smaller group of respondents was engaged in one full-time employment (*N*=148; 35.9%). Only one teacher worked less than half of a full-time job (0.2%). The characteristics of the study group are presented in Table 1.

**Table 1:** Characteristics of the study participants.

Variable			Gender				Value of *p*	Total		Female		Male	
	*n*	%					
	*n*	%	*n*	%			
Population (N)	412	100	293	71.1	119	28.9	Chi=15.195 *df*=4<0.005
Age (years)							
< 26	9	2.2	4	1.4	5	4.2	
26–35	82	19.9	49	16.7	33	27.7	
36–45	160	38.8	116	39.6	44	37.0	
46–55	118	28.7	96	32.8	22	18.5	
> 55	43	10.4	28	9.5	15	12.6	
Seniority (years)							Chi=14.261 *df*=4<0.01
< 5	32	7.8	18	6.1	14	11.8	
5–10	49	11.9	29	9.9	20	16.8	
11–15	88	21.4	57	19.5	31	26.0	
16–20	80	19.4	60	20.5	20	16.8	
> 20	163	39.5	129	44.0	34	28.6	
Form of employment (full-time contract)							Chi=12.385 *df*=4<0.01
< 0.5	1	0.2	1	0.3	0	0	
0.5	14	3.4	5	1.7	9	7.6	
> 0.5 – < 1	19	4.6	13	4.4	6	5.0	
1	148	35.9	115	39.3	33	7	
> 1 – < 1.5	152	36.9	106	36.2	46	38.7	
1.5	51	12.4	39	13.3	12	10.1	
> 1.5	27	6.6	14	4.8	13	10.9	

*p – test χ^2^.*

Type D personality was found in 30.1% (*N*=124) of the respondents, intermediate type in 36.6% (*N*=151), and non-type D personality in 33.3% (*N*=137). The intermediate personality was the most common among women – 38.6%; (*N*=113). The dominant personality type among men was non-type D (*N*=44; 37.0%). In the surveyed population, distressed personality was the least frequent (*N*=87; 29.7% – women and *N*=37; 31.1% – men). No statistically significant differences were found between the occurrence of a distressed personality between men and women (Table 2).

**Table 2:** Type of personality among respondents.

Type of personality			Gender				Value of *p*	Total		Female		Male	
	*n*	%					
	*n*	%	*n*	%			
Type D personality	124	30.1	87	29.7	37	31.1	Chi=1.768 *df*=2=0.413
Intermediate personality	151	36.6	113	38.6	38	31.9	
Non-type D personality	137	33.3	93	31.7	44	37.0	

*p –* test *χ^2^.*

Using the PSS-10 questionnaire, it was found that the median severity of stress on the sten scale in the entire group of respondents was six (lower quartile 4; upper quartile 7). After breakdown by gender, it was shown that the median severity of stress for men was 5 (lower quartile 4; upper quartile 6), and for women, it was 6 (lower quartile 4; upper quartile 7). The analysis of the severity of stress depending on the personality type showed that most often high levels of stress were found in teachers with type D personality (*N*=66; 45.5%; Table 3).

**Table 3:** Severity of stress depending on the personality type in surveyed population.

Type of personality	Stress severity category according to PSS-10						Value of *p*
	Low *N* =120		Moderate *N* =147		High *N* =145		
	*n*	%	*n*	%	*n*	%	
Type D personality	11	9.2	47	32.0	66	45.5	Chi=13.783 *df*=2<0.001
Intermediate personality	43	35.8	51	34.7	57	39.3	
Non-type D personality	66	55.0	49	33.3	22	15.2	

*p –* test *χ^2^*.

The prevalence of insomnia was estimated in the study group of teachers. Insomnia was diagnosed among *N*=204; 49.5% of teachers, which was significantly more frequent in women *N*=161; 54.9% than in men *N*=43; 36.1%, *p*<0.0005. Among teachers with intermediate personality (39%) and type D (39%) and those describing their level of stress as high (47.6%) and moderate (34.8%), the occurrence of insomnia was more frequent (*p*<0.001; Table 4 **)**.

**Table 4:** The incidence of insomnia among the studied group of teachers, including the type of personality and the level of perceived stress.

Variable	Insomnia *N* =204		Non-insomnia *N* =208		Value of *p*
	*n*	%	*n*	%	
Type of personality Type D personality	80	39	45	22	Chi =26.314 *df*=2<0.001
Intermediate personality	79	39	71	34	
Non-type D personality	45	22	92	44	
Level of perceived stress					Chi=35.893 *df*=2<0.001
Low	36	17.6	84	40.4	
Moderate	71	34.8	76	36.5	
High	97	47.6	48	23.1	

*p –* test *χ^2^.*

In the current study, the personality type, female gender, short and longest work experience, and the coexistence of depression had the greatest impact on the occurrence of insomnia. The incidence of insomnia did not change significantly with the age and seniority of teachers (*p*>0.05). The results are presented in Table 5 using logistic regression and odds ratio (*OR*).

**Table 5:** Factors determining the occurrence of insomnia in the group of surveyed teachers.

Dependent variable	Predictor	Predictor value (*N*)	*OR* (95% CI)	Value of *p*
Insomnia	Gender	Male (*N*=43)	1	< 0.01
		Female (*N*=161)	2.15 (1.38–3.34)	
	Age (years)	< 26 (*N*=2)	1	> 0.05
		26–35 (*N*=40)	3.33 (0.65–17.01)	
		36–45 (*N*=72)	2.86 (0.57–14.21)	
		46–55 (*N*=70)	4.95 (0.98–24.91)	
		> 56 (*N*=20)	3.04 (0.56–16.36)	
		<5 (*N*=9)	1	
	Seniority (years)	5–10 (*N*=27)	3.13 (1.20–8.14)	= 0.01
		11–15 (*N*=42)	3.13 (1.20–8.14)	= 0.05
		16–20 (*N*=38)	2.33 (0.97–5.60)	= 0.06
		> 20 (*N*=88)	2.99 (1.30–6.87)	= 0.009
	Type of personality	Non- type D personality (*N*=45)	1	< 0.001
		Intermediate personality (*N*=80)	2.30 (1.42–3.71)	
		Type D personality (*N*=79)	3.58 (2.15–5.98)	
	Depression	Yes (*N*=77)	6.81 (3.84–12.05)	< 0.0001
		No (N=127)	1	

Insomnia symptoms – *N*=204. *OR* – odd ratio; CI = confidence interval; and *p* – logistic regression.

Applying the Beck Depression Scale, it was found that depressive behaviors were present in *N*=94; 22.8% of teachers, significantly more often in women *N*=77; 26% than in men *N*=17; 14%, *p*<0.01.

Type D personality (52%) and high level of stress (79.8%) significantly determined the occurrence of depression (*p*<0.001). Depression was the least common among teachers with low level of stress and a non-type D personality (Table 6).

**Table 6:** The prevalence of depression among the studied group of teachers, including type of personality and the level of perceived stress.

Variable	Depression *N* =94		Non-depression *N* =318		Value of *p*
	*n*	%	*n*	%	
Type of personality					Chi=1.85 *df*=2<0.001
Type D personality	49	52	76	24	
Intermediate personality	39	42	111	35	
Non-type D personality	6	6	131	41	
Level of perceived stress					Chi =109.857 *df*=2<0.001
Low	2	2.1	118	37.1	
Moderate	17	18.1	130	40.9	
High	75	79.8	70	22.0	

*p –* test *χ^2^.*

The factors determining the occurrence of depression in the studied group of teachers include gender, personality type, and the occurrence of insomnia. Both the age and seniority as well as the employment level did not affect the incidence of depression. The results are presented in Table 7 using logistic regression and *OR*.

**Table 7:** Factors determining the occurrence of depression in the group of surveyed teachers.

Dependent variable	Predictor	Predictor value (*N*)	*OR* (95% CI)	Value of *p*
Depression	Gender	Male (*N*=17)	1	< 0.01
		Female (*N*=77)	2.13 (1.20–3.80)	
	Age (years)	<26 (*N*=0)	-	> 0.05
		26–35 (*N*=18)	1	
		36–45 (*N*=31)	0.59 (0.30–1.13)	
		46–55 (*N*=38)	1.17 (0.60–2.24)	
		> 56 (*N*=7)	1.44 (0.59–3.79)	
	Seniority (years)	<5 (*N*=5)	1	> 0.05
		5–10 (*N*=10)	1.38 (0.42–4.50)	
		11–15 (*N*=19)	1.48 (0.50–4.38)	
		16–20 (*N*=15)	1.24 (0.41–3.77)	
		> 20 (*N*=45)	2.05 (0.74–5.67)	
	Type of personality	Non-type D personality (*N*=6)	1	< 0.0001
		Intermediate personality (*N*=39)	7.60 (3.10–18.62)	
		Type D personality (*N*=49)	14.26 (5.83–34.87)	
	Insomnia	Yes (*N*=77)	3.01 (1.70–5.33)	< 0.001
		No (*N*=17)	1	

Depression symptoms – *N*=94. *OR* – odd ratio; CI = confidence interval; and *p* – logistic regression.

## Discussion

Currently, many scientific studies indicate that type D personality has a significant role in the pathogenesis of psychosomatic diseases. Taking into account, the specificity of the teaching profession (characteristic stressors and workloads) and the fact that temperament and personality determine the level of experienced stress, defining the relationship between type D personality, the level of perceived stress, and the occurrence of depressive disorders among teachers would be of great interest.

Our results have shown that 30.1% (*N* =124) of teachers had type D personality (women – 29.7%; men – 31.1%), which means that the characteristics of a distressed personality could have a negative impact on efficiency of work of about one-third of the respondents. This result is consistent with the results of various studies on the general population – for example, it has been shown that the prevalence of type D personality in British adults ranged from 29 to 38.5% (Williams et al., 2008; Borkoles et al., 2010). Similarly, in the general population of Germany, the prevalence of type D personality was 31% (Grande et al., 2010). Significantly, lower values than those obtained in the authors’ study were found in the population of European and Asian students: 12.5–25.9% (Pedersen et al., 2009; Meulenbroek et al., 2010; Polman et al., 2010; Lee et al., 2012; Condén et al., 2013b).

The analysis of personality type by occupation showed that teachers belong to a group where the prevalence of type D personality is moderate. Slightly lower rates were found in the study of female teachers in Belgium and Hungary (28.4%; Thomas et al., 2006) and in a professional group of psychiatrists and nurses in Poland (27.8%; Oginska-Bulik, 2006). A higher percentage of distressed personality was reported among nurses in South Korea – 36.8% (Kim et al., 2017). The disparity of the results may be influenced by sex, age, and size of the studied population, socioeconomic conditions, lifestyle, working and living conditions, and cultural differences.

Stress is an inseparable part of working life in many occupations. This is specifically true in the teaching profession. The inconvenience associated with current study requires exceptional attention, both due to the escalation of the occurrence of occupational stress and the serious social consequences that it can lead to. This study shows that about 36% of the respondents experienced high and medium levels of stress. The feeling of severe stress was statistically more common among female teachers and those with type D personality (45.5%). Research by other authors carried out in the same professional group shows the differentiated stressiveness of this profession – a high level of stress was declared by 20–66% of the respondents (Fontana and Abouserie, 1993; Smith et al., 2000; Denhere, 2011; Hasan, 2014).

One of the consequences of exposure to stress can be insomnia. A bulk of evidences indicates that stress increases the hypothalamic–pituitary–adrenal (HPA) axis activity due to enhanced secretion of corticotropin-releasing hormone initiating a cascade of events that culminate in the release of cortisol from the adrenal cortex which in turn promotes sleep fragmentation (Holsboer et al., 1988; Buckley and Schatzberg, 2005), yet this same sleep disturbances elevate plasma cortisol levels (Spath-Schwalbe et al., 1991). These two facts suggest an existence of model for initiation and perpetuation of a vicious circle of chronic insomnia. Notably, depression is also stress-related disorder and that HPA-overactivity contributes to its pathophysiology (Bosch et al., 2012). Remarkably, insomnia accounts for a cardinal feature of major depression and may precede the onset of this psychiatric disorder (Breslau et al., 1996; Chang et al., 1997). Also, clinical observations add some evidences for reciprocal interconnectedness; treatment of comorbid insomnia improves depression (Luik et al., 2017), conversely antidepressant medication result in sleep quality improvement in patients with insomnia as a result of depression (Saletu-Zyhlarz et al., 2002; Winokur et al., 2003).

In the studied group of teachers, the prevalence of insomnia was high and amounted to 49.5%, with women bearing a significantly higher burden than men. As for the personality type, both the intermediate and type D appeared to be significant (*p*<0.001) factors in determining the occurrence of sleep disorders. High levels of stress were significantly more frequent in teachers with insomnia (47.6%) and depression (79.8%). Our results are consistent with similar studies performed by other authors. They indicate that short-term insomnia affects from 30 to 50% of people in the general population (Ellis et al., 2012; Sateia et al., 2017). It should be emphasized that difficulties with falling asleep or maintaining sleep were found in 20–30% of the population in Poland (Szelenberger et al., 2007). This is important because chronic insomnia is associated with adverse health effects, including disturbance of the entire organism homeostasis and deterioration of quality of life. Research conducted in the American population has shown that 28.3% of people sleep less than 6h a day. The correlation between insomnia and coexistence of depression and other chronic diseases, including cardiovascular diseases and diabetes, was also confirmed (Krueger and Friedman, 2009). Similar results were obtained for the general populations in Portugal (Ohayon and Paiva, 2005) and Greece (Paparrigopoulos et al., 2010). Consistent with our results, those studies also found that prevalence of insomnia is more common among women compared to men. Although most publications indicate a positive correlation between age and insomnia, no notable relationship was found in the authors’ research.

Sleep disturbances among teachers have become the subject of interest of researchers in Poland and around the world. Similar results in terms of prevalence of insomnia symptoms to the results of this study were confirmed, for example, in the professional group of Portuguese teachers – one or several symptoms of the disorder were declared by 40.6% of the respondents, especially women (Pereira et al., 2014). In turn, Glogowska-Gruszka and Josko-Ochojska (2014) showed the presence of at least one symptom of insomnia in 74.33% of women and 71.26% of men. Our results contrast with both the above-cited studies and those carried out among teachers in Hong Kong, where only 8% of the respondents stated that they experienced insomnia frequently or very often (Jin et al., 2008). Such extreme differences in the results of the cited studies can be explained by the different diagnostic tools used and the variation in the definition of the concept of insomnia.

It is noteworthy that currently, there is a limited number of publications relating to the connection between type D personality with insomnia. Research conducted among teenagers in Sweden showed that distressed personality occurs in 12.5% of respondents (statistically more often in women than in men). Moreover, adolescents with type D personality showed a four times higher risk of sleep disorders than people with non-type D. The authors explain this dependency by a high level of experienced stress and the use of ineffective coping techniques (Condén et al., 2013a). A similar analysis was carried out among the general population of Great Britain. Results provide evidence that type D personality is related to symptoms of insomnia, both as a categorical and dimensional construct. Respondents characterized as type D reported greater symptoms of insomnia relative to non-type D individuals (Akram et al., 2018). Present study also found that among teachers with type D and intermediate personality, insomnia was observed significantly more often.

Insomnia is a documented risk factor for the occurrence of depression, which intensifies the negative attitude toward work and duties, and adversely affects the style and efficiency of work. Depression is one of the most widespread mental disorders in the world, generating significant health costs. It is a disease that causes individual losses manifested by deterioration of interpersonal relationships and difficulties in performing daily activities, such as study and work. According to the literature, depression is more common in the South America and South Asia than in Western Europe. It is also more characteristic, as in the case of insomnia, in women than in men (Ferrari et al., 2013). In the present study, using the Beck Depression Scale, it was found that depressive behaviors were presented in 22.8% of teachers, significantly more often in women than in men and in teachers with type D personality (52%).

Psychophysical burden in the work environment is one of the factors determining the stressfulness of some professions. Research shows that social workers and nursing homes, catering workers (waitresses, female kitchen staff) were more likely to suffer from depression (11–15%) compared to the general population (7%). Social workers who deal with human suffering, poverty, and various pathologies on a daily basis, with little positive reinforcement, also showed high rates of depression. In the same study, it was confirmed that teachers, nurses, secretarial staff, skilled laborers, accountants, and cashiers also suffered from depression (Kliszcz et al., 2004).

The scientific literature provides a wide variety of data on the prevalence of depression in the teacher population. Desouky and Allam (2017) using the Beck questionnaire found that symptoms of depression were present in 23.3% of Egyptian teachers; more often in women, people over 40, with low wages, high qualifications and excess responsibilities. Analogous to our results, the authors also found a positive correlation between the intensity of stress and depressive behaviors. Such a relationship becomes understandable when we consider the fact that research indicates that depression is a stress-induced structural disorder of the central nervous system involving atrophy of brain neurons as a result of reduced expression of certain neurotrophic factors necessary for keeping nerve cells alive (e.g., a cerebral neurotrophic factor). These dependencies are explained by the hypothesis of neuroplasticity disorders, which assumes the existence of a dysfunction of the HPA axis responsible for the biological response of the organism to stress factors.

The prevalence of depression is very diverse. Scientific reports indicate that symptoms of depression occur in 19.4% (Great Britain) – 50.3% (Brazil) of the surveyed teachers depending on the country of origin – (Gasparini et al., 2006; Carlotto and Câmara, 2015; Bianchi et al., 2016; Kidger et al., 2016). Large discrepancies among these results can be explained by the difference in research methodologies utilized. These include different scales for assessing depression symptoms, adopting a different cutoff point for healthy people in Beck’s questionnaire, differences in cultural, socioeconomic conditions, and access to the healthcare system.

The literature also includes several studies explaining the relationship between the distressed personality (type D) and the occurrence of depression. For example, it has been shown that among dietetics students with type D personality, depression occurred significantly more often than in non-type D (43.08% vs. 7.14%; Nowak et al., 2016). Most of these observations are consistent with research by other authors; a similar prevalence of depressive disorders was found in Indian medical students (*N*=150; 45.3%). It is noteworthy that 36% of those students with depression have confirmed type D personality (Gupta and Basak, 2013). Population studies conducted in Germany on a group of 2,459 healthy respondents showed that 777 of them (31.1%) are people with type D personality. It also turned out that this personality type was a predictor of depression (12.9%), panic and anxiety (10.4%), a sense of low health status, insufficient control over own health (*p*<0.0001), and alcohol dependence (*p*=0.037; Michal et al., 2011).

## Potential Limitations and Future Directions

This study has several potential limitations. First, the methodology used in this study allowed for a generalized presentation of the percentage of teachers with type D personality without controlling for other concomitant variables, such as the type of school, marital status, or place of residence. These variables may be important due to several factors stemming from differentiation of professional duties. These factors include type of institution (primary, secondary, or higher education), personal attitude toward students, and constraints imposed by marital status or other specificity of working conditions, such as rural vs. urban schools. This study focused on showing general indicators for the variables assigned.

Moreover, the respondents were not able to report health problems during the study. Type D personality is often the subject of research in populations affected by a specific disease, but the aim of this study was to indirectly demonstrate the psychological predispositions of teachers to perform their professional duties and to indicate the real limitations with which this type of personality is associated.

It should be emphasized that a standardized questionnaire was used to assess the prevalence of insomnia, and no objective study, such as polysomnography, was conducted. The use of the questionnaire may be the cause of the increased incidence of insomnia, but it is worth noting that the basic criterion for the diagnosis of many depressive disorders, including insomnia, is the subjective feelings of patients. Nonetheless, the final diagnosis must always be made by a specialist.

Another limitation of this study is the small group of male respondents. Teaching profession in Poland is largely feminized, which did not make it possible to survey comparable size groups of male and female respondents. However, it is believed that the sample size for the male respondents was sufficiently large to draw meaningful conclusions.

Ultimately, the present study is based on simple statistical methods to provide basic descriptive and analytical comparison among different groups. The results will be the basis for a further study in near future.

## Conclusion

It should be noted that type D personality occurs in a great percentage of teachers (30.1%). Teachers with type D personality experienced a much higher level of stress and more often suffered from insomnia and depression. Thus, the personality type of a person in teaching profession is a major factor that modulates their health. An important task for the scientific community may be the frequent examination of the teachers, and in particular awareness for self-health management. The teaching community must be alerted and informed of the features that pose a threat to harmonious and satisfactory professional development. If deemed necessary, preventive activities and interventional measures aimed at ensuring safe and healthy work environment may be imposed.

## Data Availability Statement

The datasets presented in this article are not readily available because the participants of this study did not agree for their data to be shared publicly. Requests to access the datasets should be directed to JD (jdomagalska@sum.edu.pl).

## Ethics Statement

Ethical review and approval was not required for the study on human participants in accordance with the local legislation and institutional requirements. The patients/participants provided their written informed consent to participate in this study.

## Author Contributions

JD: substantial contributions to the research concept and design, acquisition of data, analysis and interpretation of data, drafting the article or revising it critically for important intellectual content, final proofreading and approval of the version for publication. PN: substantial contributions to the research concept and design, drafting the article or revising it critically for important intellectual content, final proofreading and approval of the version for publication. MoR and MeR: final proofreading and approval of the version for publication. All authors contributed to the article and approved the submitted version.

## Funding

This study was supported by Medical University of Silesia, Katowice, Poland.

## Conflict of Interest

The authors declare that the research was conducted in the absence of any commercial or financial relationships that could be construed as a potential conflict of interest.

## Publisher’s Note

All claims expressed in this article are solely those of the authors and do not necessarily represent those of their affiliated organizations, or those of the publisher, the editors and the reviewers. Any product that may be evaluated in this article, or claim that may be made by its manufacturer, is not guaranteed or endorsed by the publisher.
